# Pulmonary Cement Embolism After Vertebroplasty

**DOI:** 10.7759/cureus.39194

**Published:** 2023-05-18

**Authors:** Muhammad K Malik, Igor Wroblewski, Amir Darki

**Affiliations:** 1 Internal Medicine, Loyola University Medical Center, Maywood, USA; 2 Cardiology, Loyola University Medical Center, Maywood, USA

**Keywords:** acute pulmonary embolism, vertebroplasty complication, pulmonary angiogram, computed tomography pulmonary angiography, submassive pulmonary embolism, pulmonary cement embolism

## Abstract

Pulmonary cement embolism (PCE) is a known complication that can occur in the setting of vertebroplasty. The majority of these cases are asymptomatic and found incidentally on imaging. There are no current management recommendations regarding PCE. We present a case of a patient who underwent vertebroplasty complicated by a symptomatic sub-massive PCE.

## Introduction

Cement extravasation is a rare but potentially severe complication after vertebroplasty and can result in pulmonary cement embolism (PCE). Most PCEs remain asymptomatic and are found incidentally on postoperative imaging [1]. PCEs however, may manifest with the same symptoms as traditional acute pulmonary embolisms such as chest pain, dyspnea, palpitations, cough, and hemoptysis [2]. The literature regarding PCE is limited to case reports and a few meta-analyses focusing primarily on the incidence of PCE. There are currently no standardized guidelines regarding the management of PCE.

## Case presentation

We present an 83-year-old female with a medical history of two myocardial infarctions treated with percutaneous coronary intervention, systemic lupus erythematosus, and osteoporosis complicated by vertebral compression fractures that were treated with five vertebroplasties. The patient was transferred to our institution for the management of a suspected PCE.

**Figure 1:**
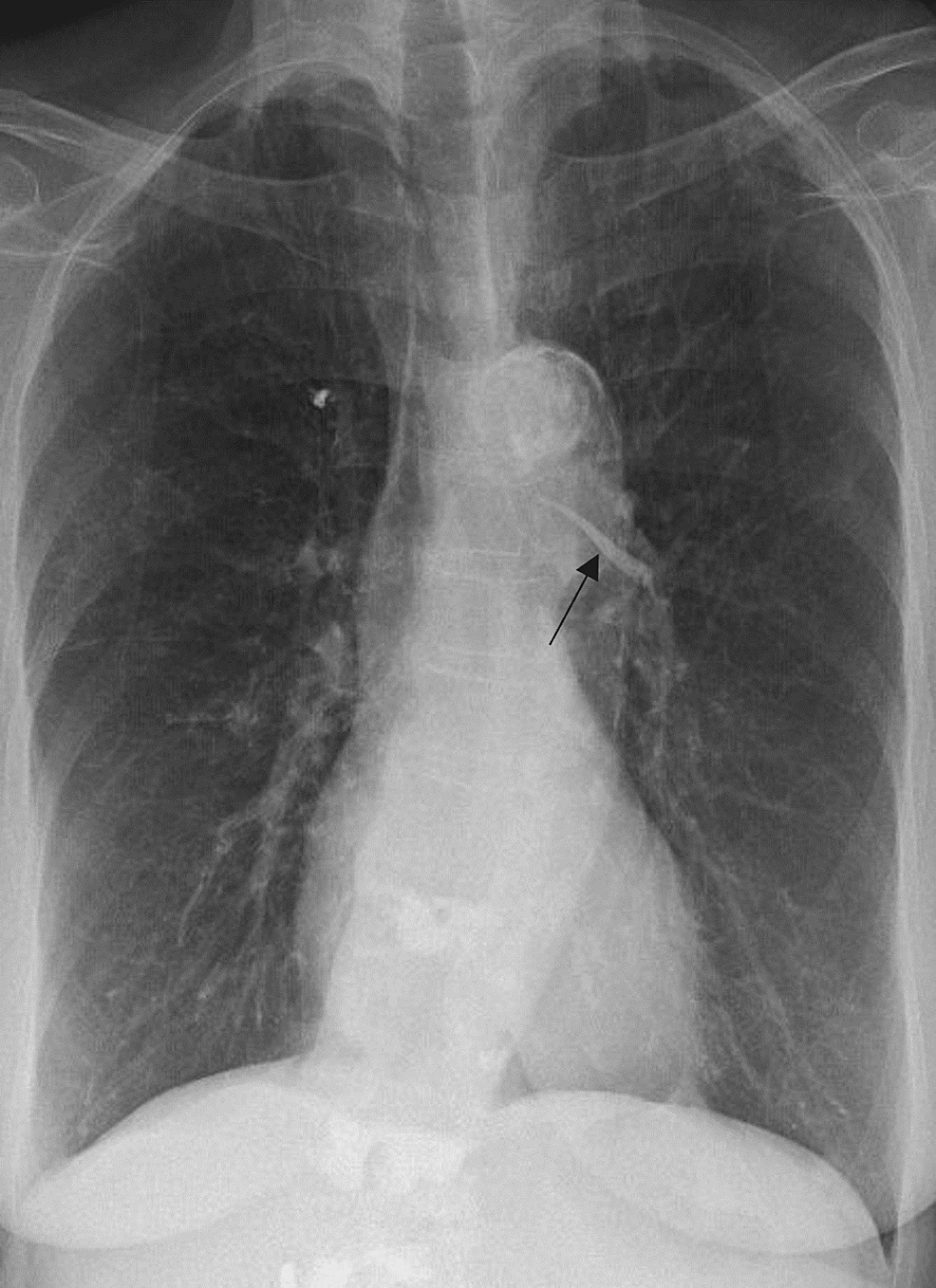
Admission chest x-ray Chest x-ray demonstrating high-density material in the pulmonary vasculature likely reflecting embolized cement is demonstrated by the black arrow.

The patient underwent her fifth vertebroplasty eight months prior to the current presentation with no reported immediate complications. She was at her baseline state of health until the day of presentation when she began to experience left-sided substernal chest pain. The patient was hemodynamically stable on initial presentation to an outside hospital. Laboratory workup was significant for a d-dimer elevation up to 736 ng/mL (normal < 500 ng/mL); her age-adjusted d-dimer cut-off was not significant (<830 ng/mL). A chest x-ray was obtained and notable for high-density perihilar material likely reflecting embolized methyl methacrylate (Figure 1).

**Figure 2:**
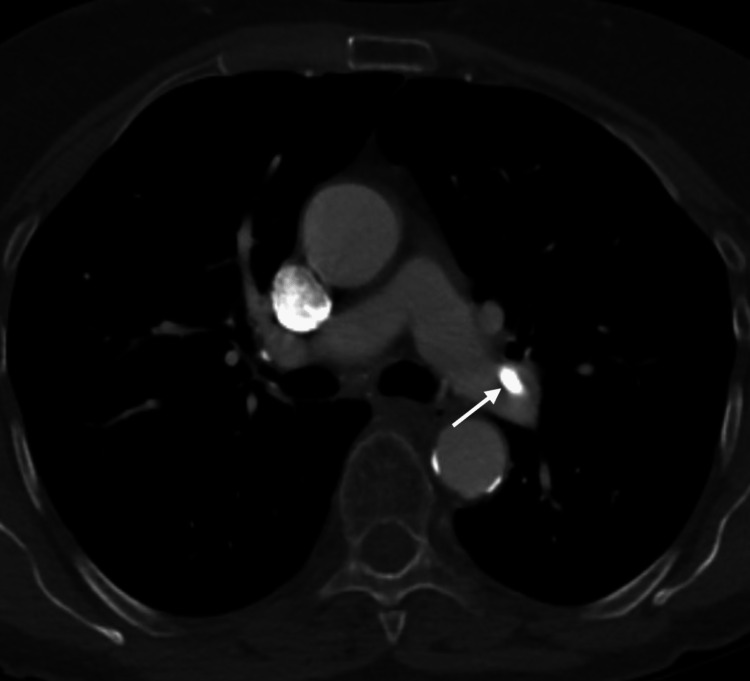
Computed tomography pulmonary angiogram High-density material representing a pulmonary cement embolism in the left main pulmonary artery is denoted by the white arrow.

Given her clinical presentation, the outside hospital obtained a computed tomography pulmonary angiogram (CTPA) which demonstrated hyperdensities; predominantly in the left main pulmonary artery extending into the left interlobar artery with additional hyperdensities in the segmental arteries of the right lung concerning a PCE (Figure 2).

**Figure 3:**
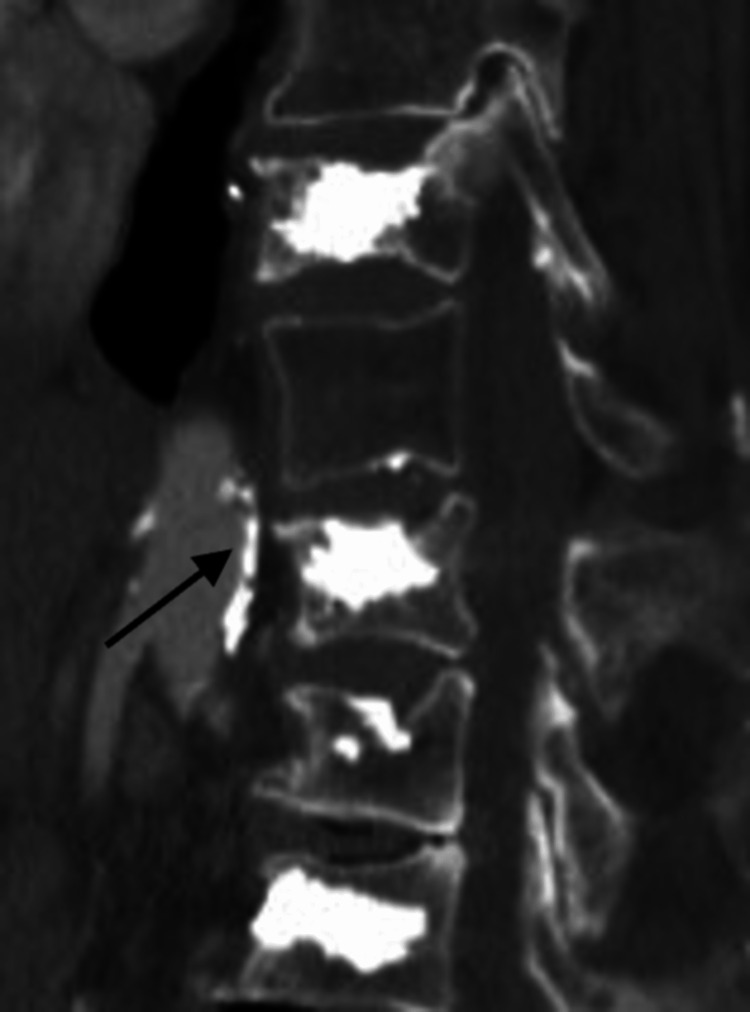
Computed tomography Hyper-dense material in the venous system adjacent to the L2 vertebroplasty location is denoted by the black arrow.

Additionally, there was high-density material adjacent to the L2 vertebral body, which was suspected to reflect cement within a paravertebral vein (Figure 3).

There was additional evidence of right ventricle (RV) strain based on RV enlargement on the CTPA with an RV/left ventricle (LV) ratio of 1.1 (normal 0.8). RV ischemia and pressure overload were also present given an elevated troponin 0.06 ng/mL (normal < 0.03 ng/mL) and brain-natriuretic peptide 131 pg/mL (normal < 100 pg/mL) confirming a diagnosis of a sub-massive pulmonary embolism. A subsequent echocardiogram demonstrated normal left and right ventricular systolic function with an ejection fraction of 65%. The tricuspid annular plane systolic excursion (TAPSE) was normal at 18 mm (normal ≥ 17 mm). There was an increase RV/LV ratio of 1.1 (normal 0.8). The patient was started on therapeutic heparin and transferred to our institution overnight.

**Figure 4:**
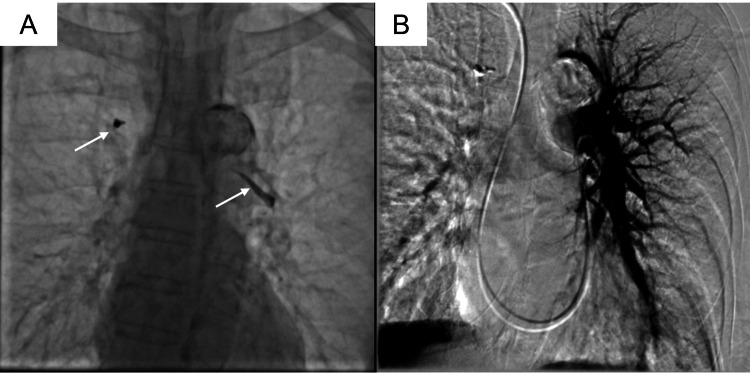
Pulmonary angiogram Fluoroscopic imaging (A) demonstrating pulmonary artery cement emboli as denoted by the white arrows can be seen. Left pulmonary angiography demonstrating no occlusive thrombotic emboli to the level of the segmental branches (B).

Doppler ultrasound of the lower extremities was performed to rule out deep venous thrombosis (DVT) as concomitant DVT has been associated with worse outcomes in patients with PE [3], and the result was negative for DVT. A right heart catheterization was performed in addition to a pulmonary angiogram. Invasive hemodynamics were as follows: mean RA 1 mmHg, PA systolic 31 mmHg, PA end-diastolic 8 mmHg, mean PA 17 mmHg, and PCWP 5 mmHg. The cardiac output was 5.1 L/min and the cardiac index was 3.0 L/min/m^2^. The pulmonary angiogram demonstrated no acute thrombotic emboli to the level of the segmental branches however the subsegmental branches were not well visualized (Figures 4A, 4B).

Given the patient’s acute chest pain with evidence of RV ischemia and pressure overload on biomarkers coupled with the inability to adequately image the subsegmental branches of the pulmonary artery on CTPA or pulmonary angiogram, we recommended three months of antithrombotic therapy. The patient was discharged on apixaban and had a three-month follow-up arranged to determine if further anticoagulation was warranted.

## Discussion

We present a case of a sub-massive PCE in the setting of vertebroplasty. The mechanism of embolization is most likely associated with the extravasation of liquid cement into the paravertebral venous system which then migrates from the azygous vein into the inferior vena cava, right atrium, RV, and finally lodging in the pulmonary artery [4].

Interestingly, the rates of asymptomatic cement extravasation in vertebroplasty can be as high as 75% based on a meta-analysis by Lee et al. [5]. According to an additional meta-analysis of 214 case reports referring to complications of vertebroplasty performed by Krueger et al, the incidence of PCE after percutaneous vertebroplasty ranges from 3.5% to 23% [6]. The majority of the cases in this meta-analysis were asymptomatic and PCEs were incidentally found on post-operative imaging. It is unclear how soon after vertebroplasty cement extravasation occurs. According to a meta-analysis performed by Ignacio and Ignacio, cement leakage appears to be temporally related to the timing of the cement injection as one study indicated that cement leakage was seen on post-op computed tomography scan in 34 out of 80 cases [4]. In a systematic review published by Wang et al., which included five observational studies and 32 case reports totaling 86 cases of PCE, symptomatic PCE was found in 85% of the case report cohort (30 out of 35 cases) [7]. Unfortunately, the rate of symptomatic PCE in the observational study cohort (51 cases) was not published in this systematic review.

There are currently no standardized guidelines regarding the management of PCE. Based on previous meta-analyses, strategies have included conservative management, heparin, coumadin, and embolectomy with the suggestion that only symptomatic PCE should be treated [4,6]. The rationale for anticoagulation in these patients stems from the fact that acrylic cement material is thought to induce activation of the coagulation cascade by stimulating the release of pro-coagulant factors by the vascular endothelium [8]; thus, the goal of anticoagulation is to help prevent further thrombotic burden. While there are no definitive studies looking at cement activating the coagulation cascade, case report data of surgical pulmonary embolectomy demonstrating cement emboli covered with thrombus may support this theory [9]. It is interesting to note that our patient had their most recent vertebroplasty eight months prior to the current presentation. As mentioned previously, the timing of cement extravasation appears to be peri-procedural. Thus, our patient’s presentation could support the notion that PCE is pro-thrombotic explaining the delayed onset of her symptoms.

Our patient was symptomatic and had evidence of right heart strain based on biomarkers and imaging. Therefore, we decided to treat her PCE medically with a direct oral anticoagulant. Three months after discharge the patient was seen in the clinic for follow-up and denied any symptoms of dyspnea on exertion or chest pain and was saturating well on room air. A repeat echocardiogram was performed which demonstrated the resolution of the RV strain. Although we were not able to find any specific guidelines regarding repeat vertebroplasty after a PCE, a thorough discussion between the physician and the patient should take place if the patient were to need another procedure in the future.

## Conclusions

Cement extravasation is a known complication after vertebroplasty and rarely can result in PCE. PCE can manifest with chest pain, dyspnea, palpitations, or hemoptysis. There are currently no standardized guidelines for the management of PCE, and previous studies have suggested only treating symptomatic PCE with heparin, coumadin, or embolectomy. We present a case of a symptomatic PCE where a direct oral anticoagulant was used for anticoagulation, adding to the limited published data on the management of PCE.
